# Mass Spectrometry Reveals Complexing Properties of Modified PNP-Lariat Ether Containing Benzyl Derivative of (S)–Prolinamine

**DOI:** 10.3390/molecules25010136

**Published:** 2019-12-29

**Authors:** Natalia Gutowska, Piotr Seliger, Jarosław Romański, Magdalena Zięba, Grazyna Adamus, Marek Kowalczuk

**Affiliations:** 1 Department of Inorganic and Analytical Chemistry, Faculty of Chemistry, University of Lodz, Tamka 12, 91403 Łódź, Poland; piotr.seliger@chemia.uni.lodz.pl; 2 Department of Organic and Applied Chemistry, Faculty of Chemistry, University of Lodz, Tamka 12, 91403 Łódź, Poland; jaroslaw.romanski@chemia.uni.lodz.pl; 3 Centre of Polymer and Carbon Materials, Polish Academy of Sciences, Marii Curie Skłodowskiej 34, 41800 Zabrze, Poland; mzieba@cmpw-pan.edu.pl (M.Z.); grazyna.adamus@cmpw-pan.edu.pl (G.A.)

**Keywords:** cyclotriphosphazene derivative, metal ion complexes, macrocycle, mass spectrometry

## Abstract

In the investigation presented here the synthesis of new lariat ether derivative obtained from the modification of tetrapyrrolidinyl-PNP-crown ether macrocycle is described. The polyheterotopic molecular coreceptor consisted of the replacement of chlorine atoms with an optically active (S)-(1-benzylpyrrolidin-2-yl) methanamine. The structure was confirmed by using elemental analysis, mass spectrometry, and NMR spectroscopy. This work covers results concerning the complexing properties of the new ligand towards Ag^+^, Cu^2+,^ Co^2+,^ Ni^2+^, and Zn^2+^ ions. The formation of non-covalent complexes of 1:1 stoichiometry with the Cu^2+,^ Co^2+,^ Ni^2+^, and Zn^2+^ ions have been confirmed by mass spectrometry. Due to the previous work and application possibilities, a large emphasis was put on the investigation of the complexation ability of lariat ether with silver (I) cation to determine stability constants by direct potentiometric method. In this case, the formation of four different forms of complexes AgL, Ag_2_L, Ag_3_L, and Ag_4_L has been proved. The observed unusual binding through the nitrogen atoms from the exocyclic substituents may provide the structural unit to build a new coordination polymers.

## 1. Introduction

In order to obtain a compound with specific practical applications, supramolecular chemistry is centered at modification of the properties of macrocycles through structural modification. Typical modifications include changing the ring size of the macrocyclic ligand, the types of donor atoms and the kinds of substituents [1,2,3].

Lariat ethers are still attractive research objects due to the possible creation of interesting structures of complexes the both with cations as well as anions. The terms “lariat ethers” refer to crown ethers having side chains attached to the crown moiety by the so-called pivot atoms (C, N, or P). The side arms provide the possibility of incorporating atoms or groups with a lone pair of electrons enabling additional cooperation with the heteroatoms of the macrocycle ring, thus providing three-dimensional coordination of the guest cation [4,5,6]. Intensive development of the lariat ethers concept has led to the preparation of the number of crown ethers, which can be used in various applications such as classical (polymer-supported catalysts PTC, reagents for separation and extraction, etc.) and highly sophisticated (the redox switches for membrane transport, synthetic cation-conducting channels, nucleotide-based molecular boxes and enzyme mimics) [7,8,9,10,11,12]. There are three known main types of lariat ethers: C-pivot lariat ethers [13,14,15], where the side arm is attached to the carbon atom, N-pivot lariat ethers [16,17,18], and finally P-pivot lariat ethers [19,20].

**Scheme 1:**
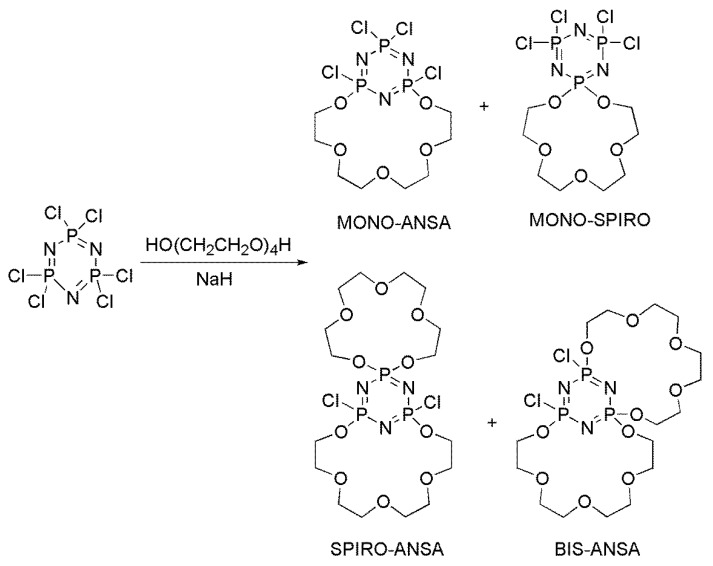
Formation of different types of macrocyclic compounds from hexachlorocyclo-triphosphazene substrate.

The subjects of this research are the P-pivot lariat ether derivatives. The versatile precursors for this type of P-pivot lariat ethers have been obtained by the reaction of hexachlorocyclotriphosphazene with tetraethylene glycol in the presence of sodium hydride (Scheme 1).

The numerous studies described in the literature on introduction different side groups indicated that oxygen and nitrogen nucleophiles cause regioselective substitution of chlorine atoms adjacent to the macrocycle. While the relevant sulphur bases such as sodium-sulphur thiolates, thiophenols, mercaptoalcohols, and dithiols, strongly prefer geminal substituting of the two chlorine atoms on the exomacrocyclic phosphorus atom from PCl_2_ group [21,22,23,24,25,26], the introduction of additional “soft” nitrogen ligands derived from amino substituent (aziridine, pyrrolidine) increases the affinity of the ligand to the larger cations (potassium, rubidium, caesium, and silver). Ligands with amino substituents are also complexing with small transition metal ions like nickel and cobalt, and cadmium cation in the case of aziridinyl derivatives [25]. Lariat ethers with alkylenediamines substituents have the complexing ability with both alkali metals, alkaline earth metals as well as transition metals. Such ligands, despite their good complexing abilities, do not exhibit high selectivity [27,28,29].

In our research group, the studies of the complexing properties of macrocyclic compounds with particular focus on P-pivot lariat ethers have been conducted for years [30,31,32,33,34]. These studies show that changes of substituents, as well as solvents used during the complexation process, affect the formation of the various structures of the complexes, especially with silver (I) cation. The studies for other cations comprise of the complexing behavior of PNP-lariat ether with other metal cations (Cd^2+^, Cu^2+^, Ca^2+^, and Pb^2+^) using ESI-MS and MS/MS techniques [35,36]. Macrocyclic derivatives of cyclotriphosphazene and their complexes were found to reveal antitumor activity as well as anti-AIDS activity [37,38,39]. Furthermore, these types of compounds are potentially useful as starting materials for the preparation of ‘pH-controlled active ion carriers’ in liquid membranes [40].

**Scheme 2:**
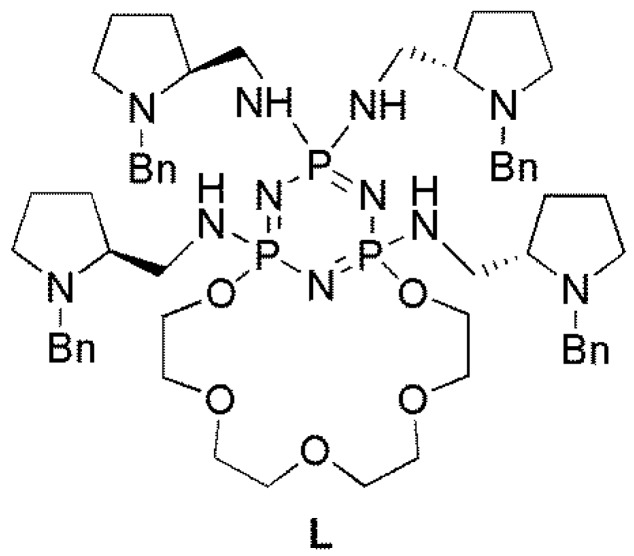
Investigated ligand L.

This article describes the new P-pivot lariat ether formed by substitution of chlorine atoms in tetrachloro-PNP-macrocycle with the N-benzyl derivative of (S)-prolinamine as side groups (Scheme 2).

This derivative was obtained from the enantiomerically pure (S)-proline, which is widely used in organic synthesis [41,42] and is the convenient substrate in the preparation of a number of derivatives with potential biological activity [43,44]. The resulting ligand L has much more binding sites in its structure that can potentially attract metal ions: (1) Oxygen donor atoms derived from the macrocyclic backbone, (2) endocyclic nitrogen atom from the cyclotriphosphazene ring, (3) exocyclic nitrogen atoms from attached substituents, and (4) benzyl groups.

To date, macrocyclic cyclotriphosphazene derivatives have not been modified by this kind of side group. Introducing them into the system will allow us to obtain compounds with a stereogenic center, which is important from the point of view of application as potential pharmacological as well as chiral recognition agents. For the obtained ligand L, the complexing capabilities towards Ag^+^, Cu^2+^, Co^2+^, Ni^2+^, and Zn^2+^ ions have been examined. The choice of such a series of ions was imposed by the possibilities of subsequent applications.

In particular silver (I) complexes are proposed for application in radioimmunotherapy [45,46,47,48], as well as an antimicrobial agent [49,50] especially for pulmonary infections related to cystic fibrosis [51,52]. Furthermore, coordination polymers based on silver (I) cations with different ligands attract attention [53,54] due to the possibility of constructing new functional materials [55,56,57,58] based on functionalized cyclotriphosphazene core.

Complexes of transition metals such as Cu^2+^, Co^2+^, Ni^2+^, and Zn^2+^ are interesting because of their use as hydrolytic metalloenzyme models [48]. Hydrolases play an important role in the biochemical processes, which can catalyze the hydrolysis of various compounds like carboxylic esters, phosphoesters, amides, nucleic acids, and peptides. The design of artificial hydrolases leads to vast application prospects in many aspects, e.g., molecular genetic tools, the degradation of toxic phosphate esters, the design of medicine, and functional materials [59,60,61,62]. Recent reports present metal complexes such as Cu^2+^, Co^2+^, Ni^2+^, Zn^2+^ with macrocycles having in its molecule nitrogen and oxygen atoms behave perfectly as an artificial hydrolase. This basic research for new derivatives of P-pivot lariat ethers will allow us to determine how stable complexes give these compounds and if possible, their use as artificial hydrolases [63].

## 2. Results and Discussion

**Scheme 3:**
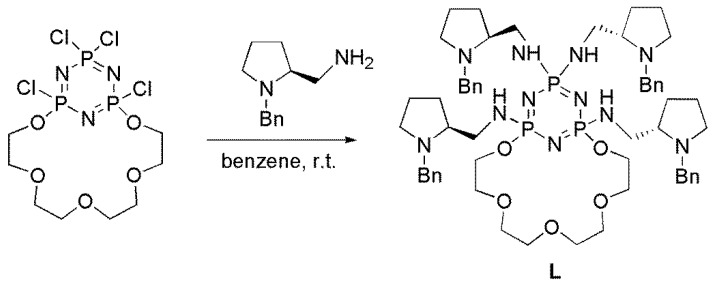
The reaction of the tetrachloro-PNP-lariat ether with (S)-prolinamine derivative.

In this paper, we reported the preparation of a new macrocyclic derivative of cyclotriphosphazene by the substitution of its reactive chlorine atoms with (S)-(1-methylpyrrolidin-2-yl) methanamine (Scheme 3).

### 2.1. Investigations of Silver Ions Complexes with the Ligand

Simple direct titration of the ligand with AgNO_3_ solution was applied for the determination of formation constants of Ag^+^ complexes with investigated compounds in acetonitrile and methanol. The analyses were performed at 298.1 ± 0.1 K with the use of 0.1 M (C_2_H_5_)_4_NNO_3_ as the supporting electrolyte. Before each measurement Nernst’s equation was verified in the following system:

Ag^0^|0.01 M AgNO_3_ + 0.09 M (C_2_H_5_)_4_NNO_3_||0.1 M (C_2_H_5_)_4_NNO_3_||

cAg + 0.1 M (C_2_H_5_)_4_NNO_3_|Ag^0^

The specific measurements were performed in the system:

Ag^0^|0.01 M AgNO_3_ + 0.09 M (C_2_H_5_)_4_NNO_3_|| 0.1 M

(C_2_H_5_)_4_NNO_3_||cL^0^ + cAg + 0.1 M (C_2_H_5_)_4_NNO_3_|Ag^0^

where cL^0^ is the initial concentration of the ligand, and cAg is the concentration of silver ions.

The experiments were triplicate for three different ligand concentrations cL^0^, which ranged from 1͘ × 10^−3^ to 1 × 10^−2^ M. The obtained reproducibility of the results was ± 0.20 mV.

The activity of the unbound silver ions was calculated on the basis of the emf values applying the Nernst equation. The stability constants were calculated using HYPERQUAD 2003 program [64]. In the case of our investigation, we made the assumption for different models of complexation equilibria and the resulting fitting parameters are presented in the final models. The presumption of the other models gave a very poor chi-square parameter or gave no reliable results at all. The determined values of stepwise stability constants were summarized in Table 1.

**Figure 1:**
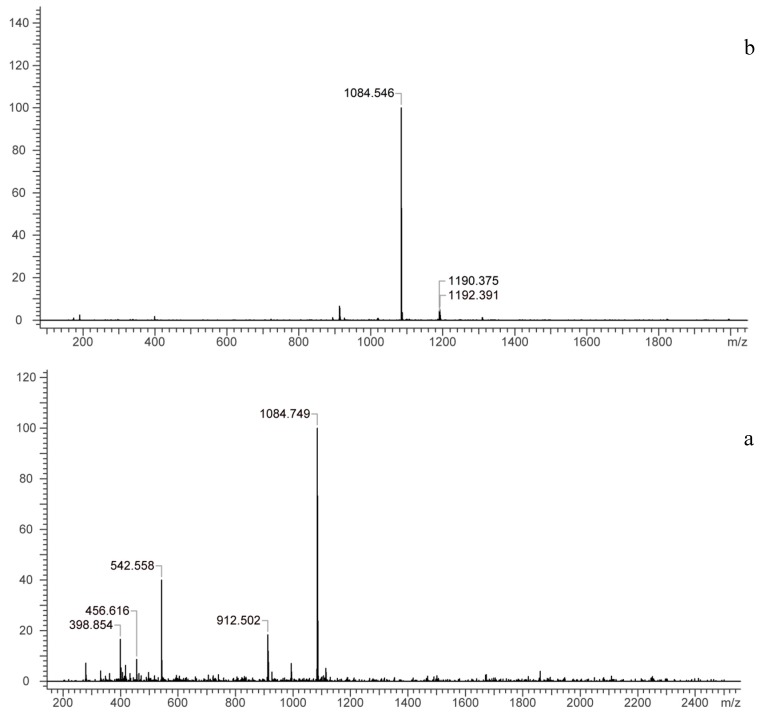
ESI-mass spectra for ligand L with AgNO_3_ in acetonitrile (**a**) and methanol (**b**).

As can be seen from Table 1, almost in all cases stability constants of investigated complexes were higher in acetonitrile solution. This tendency to form complexes with higher stability constants with macrocyclic cyclotriphosphazene derivatives occurs for the first time. Usually, other derivatives were characterized by higher stability constants in methanol [31]. The much easier formation of the complexes in acetonitrile than in methanol was confirmed by MS analysis (Figure 1). On MS spectra in methanol (Figure 1b), we did not see signals indicating the various forms of the complex, whereas in acetonitrile (Figure 1a) ligand signals with silver ions were immediately visible (*m*/*z* = 1192). This was probably related to the strong solvation of the ligand by methanol. Solvent molecules will more easily penetrate into the polyether part, to which access is facilitated, as they are not heavily hindered by large substituents. Thus, the complex formed with a 1: 1 stoichiometry in methanol, which is formed with exocyclic nitrogen atoms from substituents, has a higher stability constant (log β = 8.61) in this solvent. The formation of further forms of complexes is probably hampered by solvent molecules. It is also worth noting that Fyles and Zeng, during the study of the formation of supramolecular complexes of cations with crown ethers, noted the electrospray ionization process can result in differential production of ions from the equilibrium mixture and comparisons between ions of differing charges or stoichiometries may not correspond to the inherent selectivity of the host in solution [65].

In the case of investigated ligand, we observed four different types of complexes: AgL (1:1), Ag_2_L (2:1), Ag_3_L (3:1), and Ag_4_L (4:1). In both solvents, the most stable form of the complex is a system where one molecule of the ligand binds the first silver ion.

**Scheme 4:**
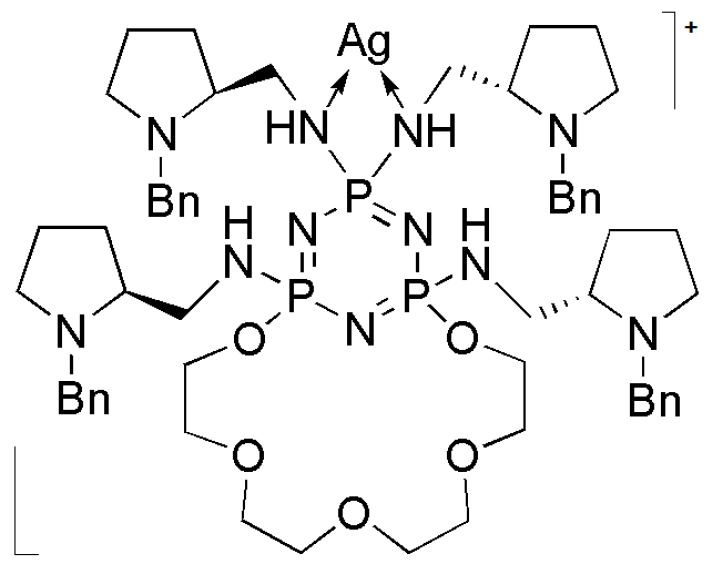
Possible structure of AgL^+^ complex.

Contrary to the previously-described PNP-lariat ethers derivatives [31,32,33,34] the sites and way of successive binding of silver ions (as confirmed by NMR and MS studies) were completely different. In the AgL system, the silver ion is bound by exocyclic nitrogen atoms derived from substituents (Scheme 4).

**Figure 2:**
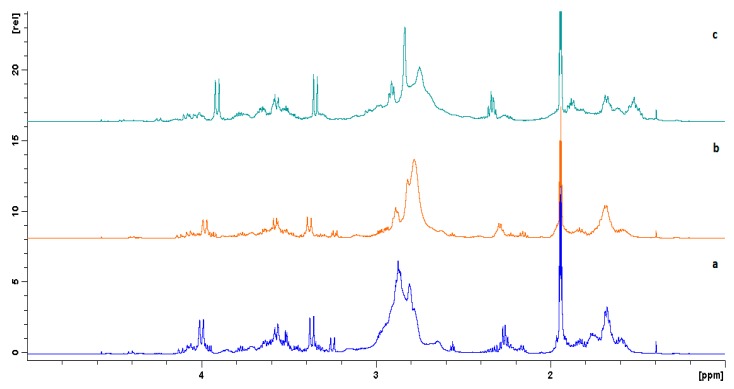
^1^H NMR spectra in acetonitrile solutions for uncomplexed ligand L-(**a**) and after adding silver ions with the Ag:L molar ratio 1:1 and 2:1-spectrum (**b**) and (**c**).

In the NMR spectra taken after the addition of the amount of silver ions corresponding to the system in a 1:1 ratio (Figure 2b), we see changes in the range of 3.10–2.65 ppm what implies that the first silver ion is binding by nitrogen atoms derived from the substituents attached to the exocyclic phosphorus atom.

**Scheme 5:**
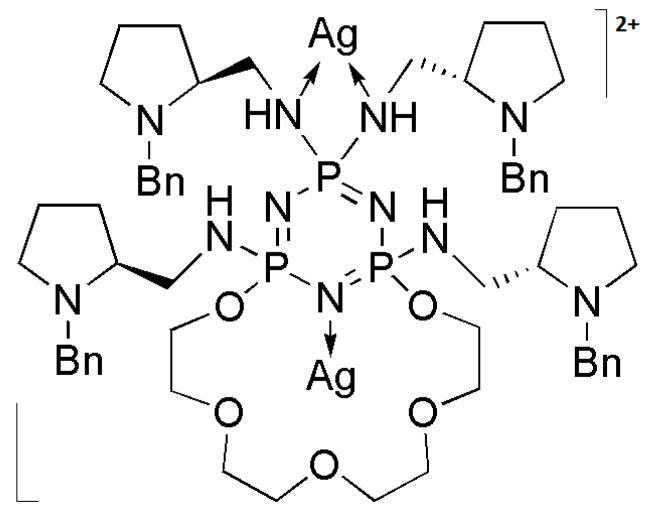
Possible structure of Ag_2_L^2+^ complex.

The second silver ion is bound by the endocyclic nitrogen atom from the cyclotriphosphazene ring. The method of binding two silver ions through ligand L is shown in Scheme 5.

Also here, the confirmation of the binding of the second silver ion is the NMR spectra (Figure 2c). The shifts of signals from the CH_2_OP group in the range of about 4 ppm are clearly visible and are similar to the ones previously described for such place of the silver ion binding [31,32,33,34]. In this stage of complexation, one can observe the shift of the signals located at about 3.30 ppm assigned to CH_2_-O fragments of the polyether ring. Such behavior suggests the additional interaction of the oxygen atoms of polyether moiety in the process of binding of the second silver ion.

**Figure 3:**
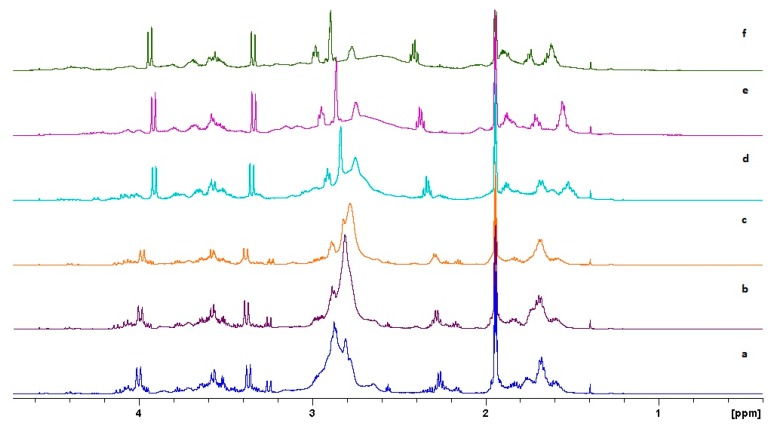
The ^1^H-NMR spectra in acetonitrile solutions for uncomplexed ligand L-spectrum (**a**) and after adding silver ions with the Ag:L molar ratio 0.5:1, 1:1, 2:1, 3:1 and 4:1-spectra (**b**), (**c**), (**d**), (**e**), and (**f**), respectively.

Subsequent silver ions in the Ag_3_L and Ag_4_L systems are bound by interaction with the π electrons of the benzyl rings in the attached substituents. In the NMR spectra ranging from 1 ppm to 5 ppm for both acetonitrile (Figure 3), no significant shifts are observed which would indicate the other site of silver ions binding.

**Figure 4:**
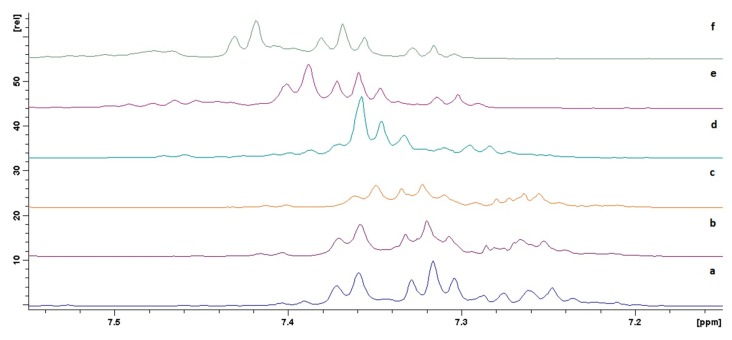
The ^1^H NMR spectra in acetonitrile solutions for free ligand L-spectrum (**a**) and after adding silver ions with the Ag:L molar ratio 0.5:1, 1:1, 2:1, 3:1, and 4:1-spectra (**b**), (**c**), (**d**), (**e**), and (**f**), respectively.

On the other hand, there are significant shifts above 7 ppm, which indicate the interaction of cation with π electrons of benzene rings (Figure 4).

For the studied ligand L, we do not observe the sandwich complex, which is related to the high steric hindrance caused by the spacious substituents.

### 2.2. Mass Spectrometry Investigations of Ligand and Its Complexes with Ag^+^ Cu^2+^, Co^2+^, Ni^2+^, and Zn^2+^ Ions

**Figure 5:**
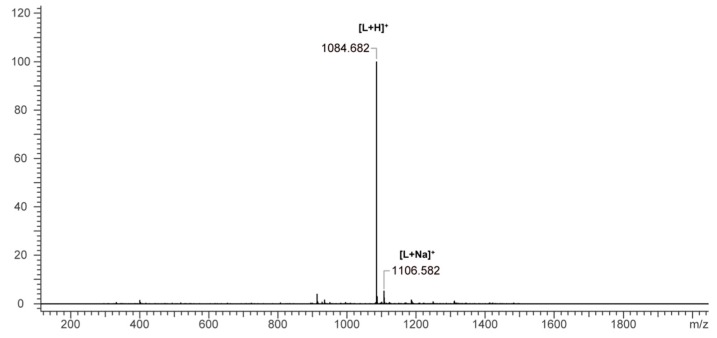
The ESI mass spectrum of investigated ligand L in acetonitrile.

**Figure 6:**
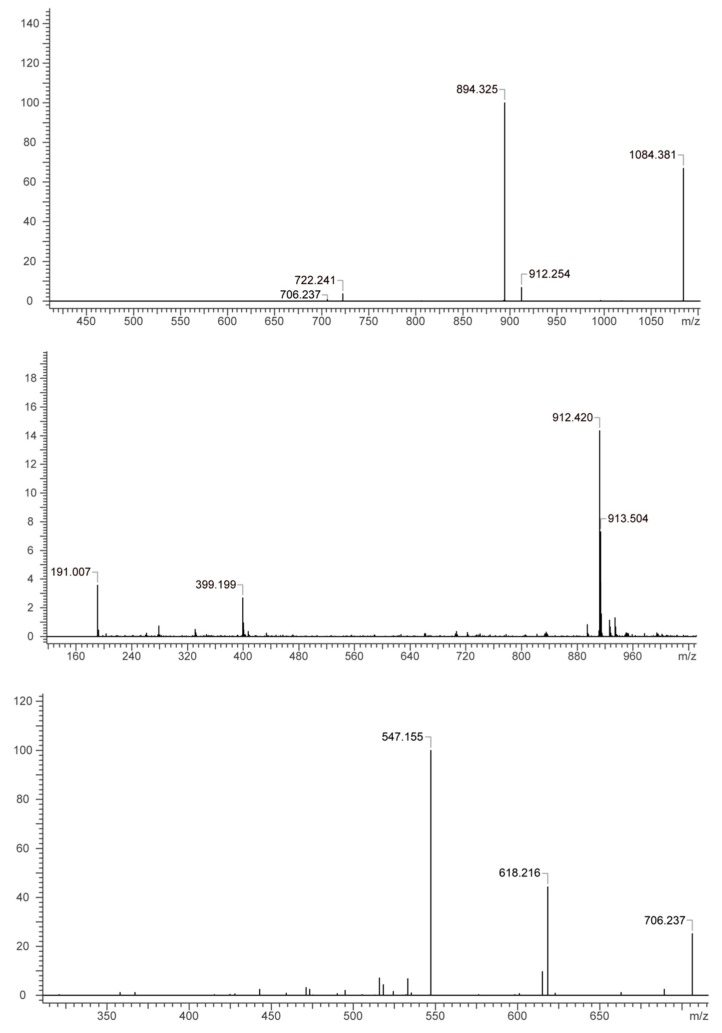
The ESI-MS/MS^2^ spectra in acetonitrile of protonated ligand [L + H]^+^ at *m*/*z* = 1084, and ESI-MS/MS^3^ of signals at *m*/*z* = 912 and 706.

**Scheme 6:**
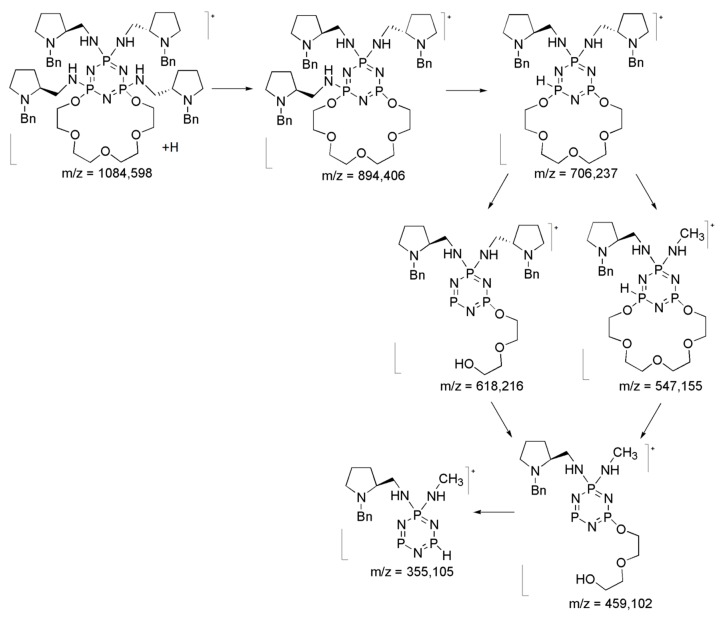
The first fragmentation pathway of the free ligand.

**Scheme 7:**
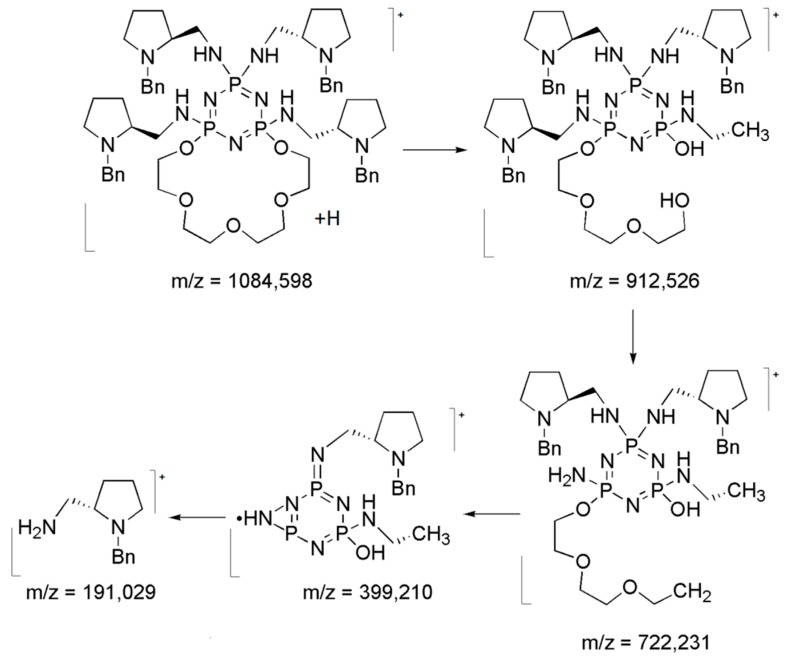
The second fragmentation pathway of the free ligand.

The ESI-MS results obtained for the basic measurements of the studied complexes are presented in Table 2. As one can see, the spectra of the investigated ligand L revealed the presence of the protonated molecule, at *m*/*z* = 1084.568 ([L + H]^+^), and a less abundant signal at *m*/*z* = 1106.582 corresponding to the sodium adduct of compound L+Na^+^ (Figure 5). The MS/MS fragmentation spectrum for the protonated ligand at *m*/*z* 1084 is presented in Figure 6. The analysis of the observed fragmentation ions shows two different series. The first fragmentation pathway passes through fragment ions with *m*/*z* 894, 706, 618, 547, 459 and 355 (Scheme 6). In the first phase, two substituents attached to endocyclic phosphorus atoms are detached, then the ring is broken down and the next fragment is removed from the substituent. The second path (Scheme 7) proceeds with the incomplete detachment of the two substituents attached to the endocyclic nitrogen atoms and further cleavage of polyether chain until a fragment of only one substituent is obtained (*m*/*z* 912, 722, 399, and 191).

After the determination of the fragmentation pattern of the protonated ligand, we took the ESI-MS spectra for the mixtures of the investigated compound with the nitrate salts of the following ions: Ag^+^, Co^2+,^ Cu^2+,^ Ni^2+^, and Zn^2+^.

**Figure 7:**
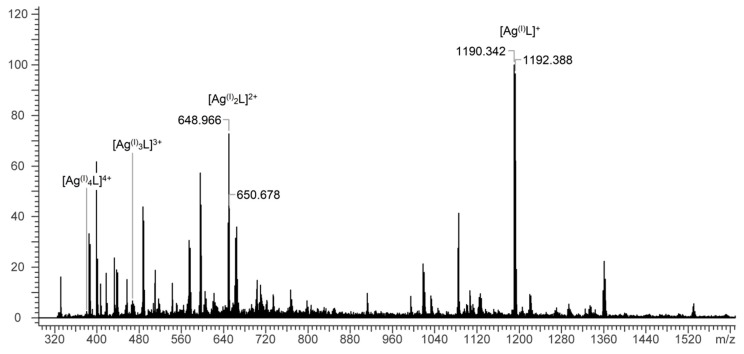
The ESI-mass spectrum of the complexes obtained by mixing the acetonitrile solutions of L with AgNO_3._

Analysis of ESI-MS spectra showed that all metal ions are bound by the investigated ligand. For a monovalent silver ion, some diverse forms of complexes were obtained, whereas, in the case of divalent cations, only complexes with a nitrate anion were observed. The confirmation of the obtained stoichiometry and ascribing to the presented forms were done on the basis of isotopic pattern comparison of the observed and simulated spectra. We can observe four different forms of complexes for Ag^+^ ions (Figure 7).

**Scheme 8:**
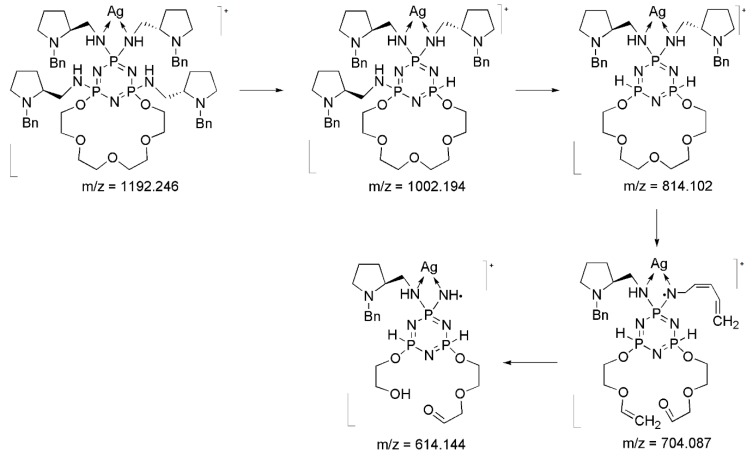
Fragmentation pathways of the [Ag(I)L]^+^ complex ion *m*/*z* 1192.

The most abundant form of the complex at *m*/*z* 1192 is derived from the [Ag^(I)^L]^+^ system. The second intensive signal is from the [Ag^(I)^_2_L]^2+^ system with two silver ions bound by the ligand. The Ag_3_L and Ag_4_L complexes were much less abundant than the ones with Ag:L ratio equal 1:1 or 2:1. In each of the observed complexes, the characteristic doubled signals with the difference of 2 Da can be noticed. Such shape of the spectrum is typical of silver complexes due to its existence in 2 isotopic forms (^107^Ag and ^109^Ag in comparable amounts). Binding of silver ions, as confirmed by NMR studies provided here, takes place through nitrogen atoms derived from substituents attached to the exocyclic phosphorus atom, an endocyclic nitrogen atom and through benzene rings. Fragmentation of the isolated signal *m*/*z* 1192 (Scheme 8) proceeds in the first stage with detaching of the substituents attached to the endocyclic phosphorus atoms (*m*/*z* 1002 and 814), followed by separation of the substituent and polyether ring fragments.

The final signal on the spectrum at *m*/*z* 1192 comes from a system in which there is a silver ion, which indicates a strong binding of this ion by the investigated ligand. 

**Figure 8:**
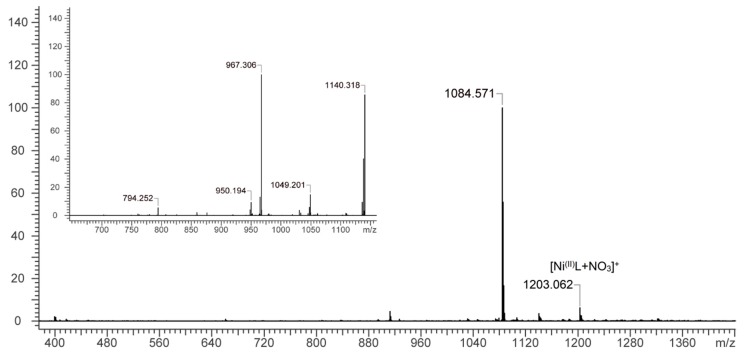
The ESI-mass spectrum of the complex obtained by mixing acetonitrile solutions of L with Ni(NO_3_)_2_ and the ESI-MS/MS^2^ spectrum of the [Ni^(II)^L + NO_3_]^+^ complex form (inset).

**Scheme 9:**
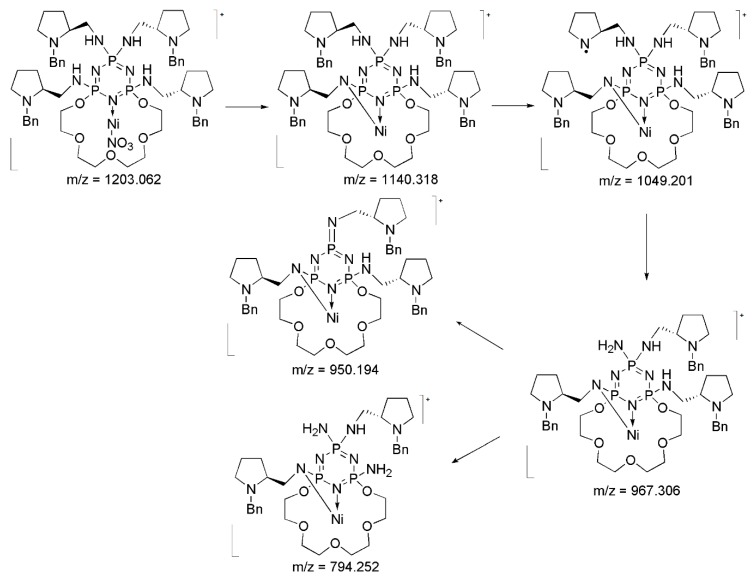
Fragmentation pathways of the [Ni^(II)^L + NO_3_]^+^ complex ion *m*/*z* 1203.

All divalent metal cations Co^2+,^ Cu^2+,^ Ni^2+^, and Zn^2+^ form only one type of complexes in which the nitrate anion is involved. The lack of other forms of complexes with divalent cations (Table 1 and Figure 8, Figure S1, Figure S2, Figure S3) for Ni^2+^, Co^2+^, Cu^2+^, and Zn^2+^ complexes, respectively) probably is related to very high steric hindrance of the parent molecule strictly connected with the branchy substituents attached to the cyclotriphosphazene subunit. Analyzing the ESI-MS spectra as well as tandem mass spectra and the subsequent fragmentation patterns of the complexes with individual divalent cations Figure 8 and Scheme 9 for [Ni^(II)^L + NO_3_]^+^ complex, and Figures S1–S6 and Schemes S1–S4 for the other investigated divalent metal ions complexes, one can see a similar tendency in the observed fragmentation routes. At the first step, the fragmentation of individual forms of complexes [ML + NO_3_]^+^ begins with the detachment of the nitrate anion, and then as a result of the ionization, the molecule loses the individual fragments of the substituents. For all doubly charged metal ions complexes, in all complexes of the ligand with divalent metal ions, the fragmentation patterns clearly show that the binding of the metal ion occurs via the endocyclic nitrogen atom. Due to the fact that in case of Ni(II), Zn(II), and Co(II) complexes the tandem mass spectra do not show the peaks related to the breaking of the polyether macrocyclic moiety of the ligand one can suppose that binding of the metal ion is also supported by additional interaction with oxygen atoms of the ligand cavity. Only in the case of Cu(II) ion, which in the experimental conditions is reduced to Cu(I), has the cleavage of the macrocyclic ring been observed, suggesting that in this case the binding of the metal ion is realized solely by endocyclic nitrogen atom of the cyclotriphosphazene subunit. Furthermore, the interaction is probably strengthened by additional stabilization by oxygen atoms from crown ether moiety (there is no cleavage of the macrocycle skeleton under the influence of the applied voltage during the experiment). Similar fragmentation pathways were also noticed in previous studies of these complexes with morpholinyl and piperidinyl substituents [35,36].

## 3. Materials and Methods

### 3.1. Materials

#### 3.1.1. For the Synthesis

(S)-(1-methylpyrrolidin-2-yl) methanamine was synthesized according to the procedure presented earlier [66]. We used Benzene (Sigma Aldrich) CHROMASOLV^®^ Plus “for HPLC”, and Hexane (POCh Gliwice, 99%). The reactions were carried out under argon atmosphere (dry).

#### 3.1.2. For the NMR Measurements

For all NMR measurements, we used deuterated solvents from Sigma Aldrich: Chloroform-d (99.8 atom % D), Methanol-d4 (99.8 atom % D) and Acetonitrile-d3 (99.8 atom % D).

#### 3.1.3. For the Potentiometric Measurement

Acetonitrile (Sigma Aldrich) for HPLC and Methanol (J. T. Baker) for HPLC were used. AgNO_3_ (Sigma Aldrich, 99.0%) and tetraethylammonium nitrate (Sigma Aldrich, 98.0%, NT) were used.

#### 3.1.4. For the MS Measurements

AgNO_3_, Cu(NO_3_)_2_, Co(NO_3_)_2_, Ni(NO_3_)_2_, and Zn(NO_3_)_2_ were analytical grade compounds from Sigma Aldrich. We used Acetonitrile for HPLC “Gradient grade” (Sigma Aldrich) and Methanol (J. T. Baker) for HPLC “Gradient Grade”. All solutions in methanol and acetonitrile (10^−3^ M each) were prepared daily prior to dilution to 10^−4^ M for mass spectrometric investigation (ligand/metal salt ratio 1:1). In each case, the freshly prepared solutions were used.

### 3.2. Characterization Techniques

#### 3.2.1. NMR Measurements

NMR spectra were recorded with Bruker (Avance III 600) instrument in CDCl_3_; chemical shifts are reported relative to solvent residual peak (^1^H: δ = 7.26 ppm [CDCl_3_]; ^13^C: δ = 77.0 ppm [CDCl_3_]; for ^31^P: δ = 0.0 ppm external reference 85% H_3_PO_4_).

#### 3.2.2. Elemental Analysis

Elemental analysis (C, H, N) was recorded using the Elementar Super Vario Micro Cube apparatus.

#### 3.2.3. Potentiometric Measurements

The potentiometric measurements were carried out with the use of Cerko microburette controlled by computer with silver electrode Ag^0^/0.01 M AgNO_3_ + 0.09 M (C_2_H_5_)_4_NNO_3_ in methanol solution as a reference electrode. The activity of the uncomplexed silver ions was calculated from the emf values by using the Nernst equation verified at the first step of the measurement procedure [34]. The stability constants were computed by using HYPERQUAD 2003 program [64]. To determine the stability constants of the complexes of investigated ligands with the other selected metal ions, the competitive potentiometric method was used [30].

#### 3.2.4. ESI-MS^n^ Analyses

The structural characterization of the complexes obtained was performed using Thermo Scientific LCQ Fleet ion trap mass spectrometer with electrospray ionization (Thermo Fisher Scientific Inc., San Jose, CA, USA). Samples of the complexes were dissolved in acetonitrile or methanol, and then the respective solutions were introduced into the ESI-MS source by continuous infusion by means of the instrument syringe pump at a rate of 10 μL min^−1^. Nitrogen was used as the nebulizing gas. The ESI source was operated at the range 3 to 3.5 kV (depending on the experiment), and the capillary heater was set to 200 °C. For ESI-MS/MS experiments, the ions of interest were isolated monoisotopically in the ion trap and were collisionally activated and the helium damping gas that was present in the mass analyzer acted as a collision gas.

The RF amplitude was set to a value that caused to decrease in the peak height of the fragmented molecular ion in the range if 70% to 30% (depending on the experiment). All experiments were performed in positive-ion mode. All the investigated fragmentation patterns were based on the results obtained from MS-Fragmenter and Spectrus Processor 2016 software (Advanced Chemistry Development, Inc., Toronto, Ontario, M5C 1B5, Canada) by ACD/Labs.

#### 3.2.5. TLC Analyses

Silica Gel 60-F254 plates (Sigma Aldrich) were used for TLC analyses.

### 3.3. Synthesis

1,3-(oxytetraethylenoxy)-1,3,5,5-tetra-[(S)-(1-methylpyrrolidin-2-yl)methanamino]-cyclotriphosphazene (L). 1,3-(Oxytetraethylenoxy)-1,3,5,5-tetrachlorocyclotriphosphazen (0.235 g, 0.0005 mole) and (S)-(1-methylpyrrolidin-2-yl) methanamine (0.760 mL, 0.004 mole) in dry benzene (50 mL) were placed in a 3-necked round-bottomed flask (100 mL) equipped with argon inlet tube. The reaction has been carried out at r.t. with continuous stirring for 72 h. TLC analysis was applied to monitor the progress of the reaction. In this case, hexane-THF (2:3) mixture was used as eluent. Then the reaction mixture was filtered and the solvent was removed under reduced pressure. The yield of the obtained ligand L: 52.6%.

^1^H NMR (600 MHz, CDCl_3_), δ(ppm): 7.32–7.17 (20H, m, HC(arom.)); 4.10 (2H, m, CH_2_OP); 3.99 (2H, m, CH_2_OP); 3.98 (4H, d, CH_2_-Ph); 3.78–3.50 (12H, m, CH_2_OC); 3.30 (4H, d, CH_2_-Ph); 3.10–3.00 (8H, m, CH_2_-NH); 2.85–2.75 (4H, m, P-NH); 2.99–2.89 (4H, m, H_2_C(5)); 2.85–2.75 (4H, m, HC(2)); 2.24–2.17 (4H, m, H_2_C(5)); 1.97–1.83 (4H, m, H_2_C(3)); 1.85–1.75 (4H, m, H_2_C(3)); 1.73–1.63 (8H, m, H_2_C(4)); ^13^C NMR (151 MHz, CDCl_3_), δ(ppm): 139.4, 128.7, 128.3, 126.9, 70.7, 70.3, 64.0, 62.1, 58.9, 58.4, 54.3, 54.1, 43.2, 42.3, 28.1, 23.0; ^31^P NMR (242 MHz, CDCl_3_), δ (ppm): 22,0 (d, P(OCH_2_)NNBn); 19.7 (t, P(NNBn)_2_, JPP = 50.0 Hz, A2B system); ESI-MS *m*/*z* = 1084.7 [M + H^+^], 1107.6 [M + Na^+^]; elemental analysis: calculated for C_56_H_84_N_11_O_5_P_3_: C 62.03; H 7.81; N 14.21; found: C 62.15; H 7.59; N 14.14.

## 4. Conclusions

In this work, the synthesis of a new macrocyclic derivative with optically active substituents in the form of a benzyl derivative of (S)-prolineamine is presented. Potentiometric and NMR studies were presented for the investigated ligand complexes with silver ions in acetonitrile and methanol. Electrochemical, NMR, and MS studies have confirmed the unexpected and promising possibilities of complex formation by a new compound. Ligand with silver ions forms four types of complexes with different stoichiometry-AgL, Ag_2_L, Ag_3_L, and Ag_4_L, with the most stable 1:1 form observed in both solvents.

Unlike similar derivatives the obtained stability constants, are higher in acetonitrile, which results from the stronger solvation of our ligand by methanol molecules. Furthermore, silver ion in the AgL complex is preferably attracted to nitrogen atoms from substituents which are attached to the exocyclic phosphorus atom. In the previously described PNP lariat ethers derivatives, binding of the first silver ion was realized by the endocyclic nitrogen atom from the cyclotriphosphazene subunit [30,31,32,33,34,35,36]. The second silver ion is bound by the endocyclic nitrogen atom of the cyclotriphosphazene ring. However, in Ag_3_L and Ag_4_L systems, there are interactions between silver ions and π electrons of benzyl rings.

MS studies confirmed the existence of complexes with Ag^+^, Cu^2+^, Co^2+^, Ni^2+^, and Zn^2+^ ions. For a monovalent silver cation, the existence of four types of complexes has been confirmed. The presence of the AgL^+^ complex with unusual binding through the nitrogen atoms from the exocyclic substituents may provide the structural unit to build a new coordination polymer. Divalent cations form only one type of complexes additionally supported by nitrate anion. Binding of Co^2+^, Ni^2+^, and Zn^2+^ ions takes place via the endocyclic nitrogen atom from the cyclotriphosphazene ring supported by oxygen atoms of polyether macrocyclic moiety. Only the case of Cu^2+^ ions reduction from copper (II) to copper (I) ion has been observed during experimental conditions and the obtained complex is only formed by endocyclic nitrogen atom from cyclotriphosphasen subunit.

The MS/MS results show that all types of complexes that are formed are stable under the conditions of applied voltage and do not undergo fragmentation with metal ion detachment.

## Figures and Tables

**Table 1:** The stability constants of the complexes of the investigated ligand with Ag^+^ ions determined by potentiometric measurements.

Complex Form	L	
	log β in Acetonitrile	log β in Methanol
Ag^+^ + L → AgL^+^	7.12 ± 0.01	8.61 ± 0.01
AgL^+^ + Ag^+^ → Ag_2_L^2+^	5.94 ± 0.01	3.83 ± 0.01
Ag_2_L^2+^ + Ag^+^ → Ag_3_L^3+^	4.28 ± 0.01	3.41 ± 0.01
Ag_3_L^3+^ + Ag^+^ → Ag_4_L^4+^	3.06 ± 0.01	1.99 ± 0.01

**Table 2:** Complexes confirmed by electrospray mass spectrometry and tandem mass spectrometry methods (ESI-MS/MS).

	L + M	L + 2M	L + 3M	L + 4M	L + M + NO_3_
Ag^+^	[Ag^(I)^L]^+^ 1192.388	[Ag^(I)^_2_L]^2+^ 648.966	[Ag^(I)^_3_L]^3+^ 468.395	[Ag^(I)^_4_L]^4+^ 378.340	---
Co^2+^	---	---	---	---	[Co^(II)^L + NO_3_]^+^ 1204.090
Cu^2+^	---	---	---	---	[Cu^(II)^L + NO_3_]^+^ 1208.414
Ni^2+^	---	---	---	---	[Ni^(II)^L + NO_3_]^+^ 1203.062
Zn^2+^	---	---	---	---	[Zn^(II)^L + NO_3_]^+^ 1209.260

## Supplementary Materials

The following are available online. Figure S1: The ESI-MS spectrum of L with Co(NO_3_)_2_, Figure S2: The ESI-MS spectrum of L with Cu(NO_3_)_2_, Figure S3: The ESI-MS spectrum of L with Zn(NO_3_)_2_, Figure S4: The ESI-MS/MS^2^ spectrum of the complex with Co^2+^ ion at *m*/*z* = 1204, Figure S5: The ESI-MS/MS^2^ spectrum of the complex with Cu^2+^ ion at *m*/*z* = 1208, Figure S6: The ESI-MS/MS^2^ spectrum of the complex with Zn^2+^ ion at *m*/*z* = 1209, Scheme S1: Fragmentation pathways of the [Co^(II)^L+NO_3_]^+^ complex ion *m*/*z* 1204, Scheme S2: The first fragmentation pathway of the [Cu^(II)^L+NO_3_]^+^ complex ion *m*/*z* 1204, Scheme S3: The second fragmentation pathway of the [Cu^(II)^L+NO_3_]^+^ complex ion *m*/*z* 1204, Scheme S4: Fragmentation pathway of the [Zn^(II)^L+NO_3_]^+^ complex ion *m*/*z* 1204.

## References

[1] Gokel G.W. (1991). Crown Ethers and Cryptands.

[2] Gokel G.W., Leevy W.M., Weber M.E. (2005). Macrocyclic Chemistry: Current Trends and Future Perspectives.

[3] Patai S., Rappaport Z. (1989). Crown Ethers and Analogs.

[4] Gokel G.W., Leevy W.M., Weber M.E. (2004). Crown Ethers: Sensors for Ions and Molecular Scaffolds for Materials and Biological Models. Chem. Rev..

[5] Gokel G.W. (1992). Lariat ethers: from simple sidearms to supramolecular systems. Chem. Soc. Rev..

[6] Brandt K., Porwolik I., Siwy M. (1997). Crown ethers and their analogs as catalysts, cocatalysts and substrates in the reactions involving ion pairs. Wiadomości Chemiczne Chemia Supramolekularna.

[7] Cooper S.R. (1992). Crown Compound-Toward Future Applications.

[8] Lehn J.M. (1993). Chemia Supramolekularna.

[9] Lindoy L.F. (1989). The Chemistry of Macrocyclic Ligand Complexes.

[10] Lehn J.-M. (1990). Perspectives in Supramolecular Chemistry—From Molecular Recognition towards Molecular Information Processing and Self-Organization. Angew. Chem. Int. Ed..

[11] Katritzky A.R., Denisko O.V., Belyakov S.A., Schall O.F., Gokel G.W. (1996). Synthesis of NewN-Pivot Lariat Crown Ethers Containing a Propylene Linkage in the Side Arm. J. Org. Chem..

[12] Komiyama M. (1993). Cyclic oligomers as highly selective catalysts. Prog. Polym. Sci..

[13] Goli D.M., Dishing D.M., Diamond C.I., Gokel G.W. (1982). Lariat ethers. VI. Evidence for intramolecular chelation in sodium and potassium cation binding by 15-crown-5, carbon-pivot lariat ethers. Tetrahedron Lett..

[14] White B.D., Arnold K.A., Gokel G.W. (1987). One- and two-armed lariat ether peptide derivatives: syntheses and cation binding properties. Tetrahedron Lett..

[15] Abbas A.A., Elwahy A.H.M. (2009). Synthesis ofC-pivot lariat ethers. J. Heterocycl. Chem..

[16] Dishong D.M., Diamond C.J., Cinoman M.I., Gokel G.W. (1983). Crown cation complex effects. 20. Syntheses and cation binding properties of carbon-pivot lariat ethers. J. Am. Chem. Soc..

[17] Schultz R.A., White B.D., Dishong D.M., Arnold K.A., Gokel G.W. (1985). 12-, 15-, and 18-Membered-ring nitrogen-pivot lariat ethers: syntheses, properties, and sodium and ammonium cation binding properties. J. Am. Chem. Soc..

[18] Elwahy A.H.M., Abbas A.A. (2008). Synthesis of *N*-pivot lariat ethers. J. Heterocycl. Chem..

[19] Brandt K., Kupka T., Drodz J., Van De Grampel J.C., Meetsma A., Jekel A.P. (1995). New dioxytetraethyleneoxy macrocyclic cyclophosphazene derivatives. Inorg. Chim. Acta.

[20] Brandt K., Porwolik I., Kupka T., Olejnik A., Shaw R.A., Davies D.B. (1995). New Lariat Ether-Type Macrocycles with Cyclophosphazene Subunits. J. Org. Chem..

[21] Davies D.B., Clayton T.A., Eaton R.E., Shaw R.A., Egan A., Hursthouse M.B., Sykara G.D., Porwolik-Czomperlik I., Siwy M., Brandt K. (2000). Chiral Configurations of Cyclophosphazenes. J. Am. Chem. Soc..

[22] Kruszyński R., Siwy M., Porwolik-Czomperlik I., Trzesowska A. (2006). A new regioselective method of macrobicyclic Schiff bases synthesis. Inorg. Chim. Acta.

[23] Maia A., Landini D., Brandt K., Penso M. (2002). Effect of the metal ion on the regiochemical outcome of substitution reactions promoted by crown-bearing cyclophosphazenes. New J. Chem..

[24] Maia A., Landini D., Brandt K., Siwy M., Gierczyk B., Penso M., Schroeder G. (2001). Functional crown ethers with chlorocyclophosphazene sub-units as anion activators and promoters of highly regioselective reactions. New J. Chem..

[25] Brandt K., Porwolik-Czomperlik I., Siwy M., Kupka T., Shaw R.A., Davies D.B., Bartsch R.A. (1999). New Molecular Receptors with Cyclophosphazene Subunits: Synthesis, Reactivity, and Structure-Property Relationships. J. Incl. Phenom. Macrocycl. Chem..

[26] Brandt K., Porwolik-Czomperlik I., Siwy M., Kupka T., Shaw R.A., Davies D.B., Hursthouse M.B., Sykara G.D. (1997). Thermodynamic vs. Supramolecular Effects in the Regiocontrol of the Formation of New Cyclotriphosphazene-Containing Chiral Ligands with 1,1′-Binaphthyl Units: Spiro vs. Ansa Substitution at the N3P3 Ring. J. Am. Chem. Soc..

[27] Brandt K., Porwolik I., Siwy M., Kupka T., Shaw R.A., Davies D.B., Hursthouse M.B., Sykara G.D. (1997). Host−Guest Complex Dependent Regioselectivity in Substitution Reactions of Chlorocyclotriphosphazene-Containing PNP-Crowns with Alkylenediamines. J. Am. Chem. Soc..

[28] Porwolik-Czomperlik I., Brandt K., Clayton T.A., Davies D.B., Eaton R.J., Shaw R.A. (2002). Diastereoisomeric Singly Bridged Cyclophosphazene-Macrocyclic Compounds. Inorg. Chem..

[29] Bartsch R.A., Lee E.K., Chun S., Elkarim N., Brandt K., Porwolik-Czomperlik I., Siwy M., Lach D., Silberring J. (2002). Structure–alkali metal cation complexation relationships for macrocyclic PNP-lariat ether ligands. J. Chem. Soc. Perkin Trans. 2.

[30] Brandt K., Seliger P., Grzejdziak A., Bartczak T.J., Kruszynski R., Lach D., Silberring J. (2001). Structure−Property Relationships of a Tetrapyrrolidinyl PNP−Lariat Ether and Its Complexes with Potassium, Sodium, and Silver Cations. Inorg. Chem..

[31] Seliger P., Sołtys N., Andrijewski G., Siwy M. (2012). Complexes of silver(i) ions with tetrapyrrolidinyl- and tetramorpholinyl-PNP-lariat ethers in acetonitrile and methanol solutions. New J. Chem..

[32] Seliger P., Andrijewski G., Siwy M., Sęk D. (2009). Silver Ion Complexes with Macrocyclic Compounds Build on the Basis of Cyclotriphosphazene Ring. Pol. J. Chem..

[33] Sołtys N., Seliger P., Andrijewski G., Siwy M. (2013). Doubly-bridged PNP-lariat ethers and their complexes with silver(i) ions in methanol solution. RSC Adv..

[34] Gutowska N., Seliger P., Andrijewski G., Siwy M., Kusz J., Małecka M. (2015). Single and double crown macrocyclic derivatives of cyclotriphosphazene as receptors of silver(i) ions. RSC Adv..

[35] Gutowska N., Pasternak B., Seliger P., Andrijewski G. (2015). Studies of the complexation behavior of tetramorpholinylo-PNP-lariat ether with Ag(I), Ca(II), Cd(II), Cu(II) and Pb(II) using Electrospray Ionization Mass Spectrometry. New J. Chem..

[36] Gutowska N., Seliger P., Andrijewski G., Adamus G., Kwiecien I. (2018). Tandem mass spectroscopy as a tool for investigation of complexes of PNP-lariat ether derivative with metal ions. J. Mass Spectrom..

[37] Brandt K., Kruszynski R., Bartczak T.J., Porwolik-Czomperlik I. (2001). AIDS-related lymphoma screen results and molecular structure determination of a new crown ether bearing aziridinylcyclophosphazene, potentially capable of ion-regulated DNA cleavage action. Inorg. Chim. Acta.

[38] Porwolik-Czomperlik I., Siwy M., Sęk D., Kaczmarczyk B., Nasulewicz A., Jaroszewicz I., Pełczyńska M., Opolski A. (2004). Synthesis and in vitro cytostatic activity of some new 1,3-(oxytetraethylenoxy)-cyclotriphosphazatriene derivatives. Acta Pol. Pharm.–Drug Res..

[39] Siwy M., Sek D., Kaczmarczyk B., Wietrzyk J., Nasulewicz A., Opolski A. (2007). Synthesis and in vitro antiproliferative activity of new 1,3-(oxytetraethylenoxy)-cyclotriphosphazene derivatives. Anticancer Res..

[40] Penso M., Maia A., Lupi V., Tricarico G. (2011). Selective O-functionalization of phenolic α-amino acids with crown ethers bearing cyclophosphazene sub-units. Tetrahedron.

[41] Jones C.A., Jones I.G., North M., Pool C.R. (1995). Asymmetric addition of organolithium reagents to imines favouring (S)-amines. Tetrahedron Lett..

[42] Hoegberg T., Raemsby S., Stroem P. (1989). Efficient stereoconservative syntheses of 1-substituted (S)-and (R)-2-aminomethylpyrrolidines. Acta Chem. Scand..

[43] Xie L., Jones G.B. (2005). Exploiting π-shielding interactions: a highly selective chiral auxiliary derived from a biogenic building block. Tetrahedron Lett..

[44] Diakos C.I., Zhang M., Beale P.J., Fenton R.R., Hambley T.W. (2009). Synthesis, characterisation and in vitro cytotoxicity studies of a series of chiral platinum(II) complexes based on the 2-aminomethylpyrrolidine ligand: X-ray crystal structure of [PtCl2(R-dimepyrr)] (R-dimepyrr=N-dimethyl-2(R)-aminomethylpyrrolidine). Eur. J. Med. Chem..

[45] Chattopadhyay S., Vimalnath K.V., Saha S., Korde A., Sarma H.D., Pal S., Das M.K. (2008). Preparation and evaluation of a new radiopharmaceutical for radiosynovectomy, 111Ag-labelled hydroxyapatite (HA) particles. Appl. Radiat. Isot..

[46] Aweda T.A., Ikotun O., Mastren T., Cannon C.L., Wright B., Youngs W.J., Cutler C., Guthrie J., Lapi S.E. (2013). The use of 111Ag as a tool for studying biological distribution of silver-based antimicrobials. MedChemComm.

[47] Aweda T.A., Cannon C., Wooley K., Youngs W., Lapi S.E. (2013). Ag-111: A radiotracer for the chemistry and biochemistry of silver antimicrobials. Abstracts of Papers of the American Chemical Society.

[48] Neves M., Kling A., Lambrecht R.M. (2002). Radionuclide production for therapeutic radiopharmaceuticals. Appl. Radiat. Isot..

[49] Klasen H. (2000). Historical review of the use of silver in the treatment of burns. I. Early uses. Burns.

[50] Hollinger M.A. (1996). Toxicological Aspects of Topical Silver Pharmaceuticals. Crit. Rev. Toxicol..

[51] Melaiye A., Simons R.S., Milsted A., Pingitore F., Wesdemiotis C., Tessier C.A., Youngs W.J. (2004). Formation of Water-Soluble Pincer Silver(I)−Carbene Complexes: A Novel Antimicrobial Agent. J. Med. Chem..

[52] Conlan L.H., Dupureur C.M. (2002). Multiple Metal Ions Drive DNA Association byPvuII Endonuclease. Biochemistry.

[53] Khlobystov A.N., Blake A.J., Champness N.R., Lemenovskii D.A., Majouga A.G., Zyk N.V., Schröder M. (2001). Supramolecular design of one-dimensional coordination polymers based on silver(I) complexes of aromatic nitrogen-donor ligands. Co-Ord. Chem. Rev..

[54] Itaya T., Inoue K. (2002). Construction of hairy-rod coordination polymers with lamellar structure by self-assembling of hexakis(4-pyridylmethoxy)cyclotriphosphazene and silver alkylsulfonates. Polyhedron.

[55] Bradshaw D., Claridge J.B., Cussen E.J., Prior T.J., Rosseinsky M.J. (2005). Design, Chirality, and Flexibility in Nanoporous Molecule-Based Materials. Accounts Chem. Res..

[56] Ainscough E.W., Brodie A.M., Davidson R.J., Otter C.A. (2008). The first coordination polymer containing a chiral cyclotriphosphazene ligand. Inorg. Chem. Commun..

[57] James S.L. (2003). Metal-organic frameworks. Chem. Soc. Rev..

[58] Evans O.R., Lin W. (2002). Crystal Engineering of NLO Materials Based on Metal−Organic Coordination Networks. Accounts Chem. Res..

[59] Mishra M., Tiwari K., Shukla S., Mishra R., Singh V.P. (2014). Synthesis, structural investigation, DNA and protein binding study of some 3d-metal complexes with *N*′-(phenyl-pyridin-2-yl-methylene)-thiophene-2-carboxylic acid hydrazide. Spectrochim. Acta Part A Mol. Biomol. Spectrosc..

[60] Sirajuddin M., Nooruddin, Ali S., McKee V., Khan S.Z., Malook K. (2015). Synthesis, spectroscopic characterization, crystal structure, DNA interaction study and in vitro biological screenings of 4-(5-chloro-2-hydroxyphenylamino)-4-oxobut-2-enoic acid. Spectrochim. Acta Part A Mol. Biomol. Spectrosc..

[61] Elton E.S., Zhang T., Prabhakar R., Arif A.M., Berreau L.M. (2013). Pb(II)-Promoted Amide Cleavage: Mechanistic Comparison to a Zn(II) Analogue. Inorg. Chem..

[62] Jiang W.D., Xu B., Liu F.A., Wang Y., Xiang Z. (2015). Catalytic Reactivity of Phenoxo-Bridged Homobinuclear Copper(II) Complexes With L-Threonine Schiff Bases As a Carboxylesterase Model. Synth. React. Inorg. Met.-Org. Nano-Met. Chem..

[63] Yu L., Li F.-Z., Wu J.-Y., Xie J.-Q., Li S. (2016). Development of the aza-crown ether metal complexes as artificial hydrolase. J. Inorg. Biochem..

[64] Gans P., Sabatini A., Vacca A. (1996). Investigation of equilibria in solution. Determination of equilibrium constants with the HYPERQUAD suite of programs. Talanta.

[65] Fyles T.M., Zeng B. (1998). On the Assessment of Complex Cation—Crown Ether Equilibria by Electrospray Mass Spectrometry. Supramol. Chem..

[66] Rispens M.T., Gelling O.J., De Vries A., Meetsma A., Van Bolhuis F., Feringa B.L. (1996). Catalytic epoxidation of unfunctionalized alkenes by dinuclear nickel(II) complexes. Tetrahedron.

