# Supplementary material for: Mass Spectrometry Reveals Complexing Properties of Modified PNP-Lariat Ether Containing Benzyl Derivative of (S)–Prolinamine
Source: Molecules. 2019 Dec 29;25(1):136. doi: 10.3390/molecules25010136 (PMC6983243; doi:10.3390/molecules25010136)
Supplement: Supplementary file 1 [file molecules-25-00136-s001.pdf]

Supporting information

Mass Spectrometry Reveals Complexing Properties of Modified PNP-lariat Ether Containing Benzyl Derivative of (S)–prolinamine

Natalia Gutowska[1,†,*], Piotr Seliger[1], Jarosław Romański[2], Magdalena Zięba[3], Grażyna Adamus [3], and Marek Kowalczuk [3,*]

[1] Department of Inorganic and Analytical Chemistry, Faculty of Chemistry, University of Lodz, 91-403 Łódź, Tamka 12, Poland
[2] Department of Organic and Applied Chemistry, Faculty of Chemistry, University of Lodz, 91-403 Łódź, Tamka 12, Poland
[3] Centre of Polymer and Carbon Materials, Polish Academy of Sciences, 41-800 Zabrze, Marii Curie Skłodowskiej 34, Poland
[†] Current affiliation: Institute of Biopolymers and Chemical Fibres, 90-570 Łódź, Marii Skłodowskiej-Curie 19/27, Poland

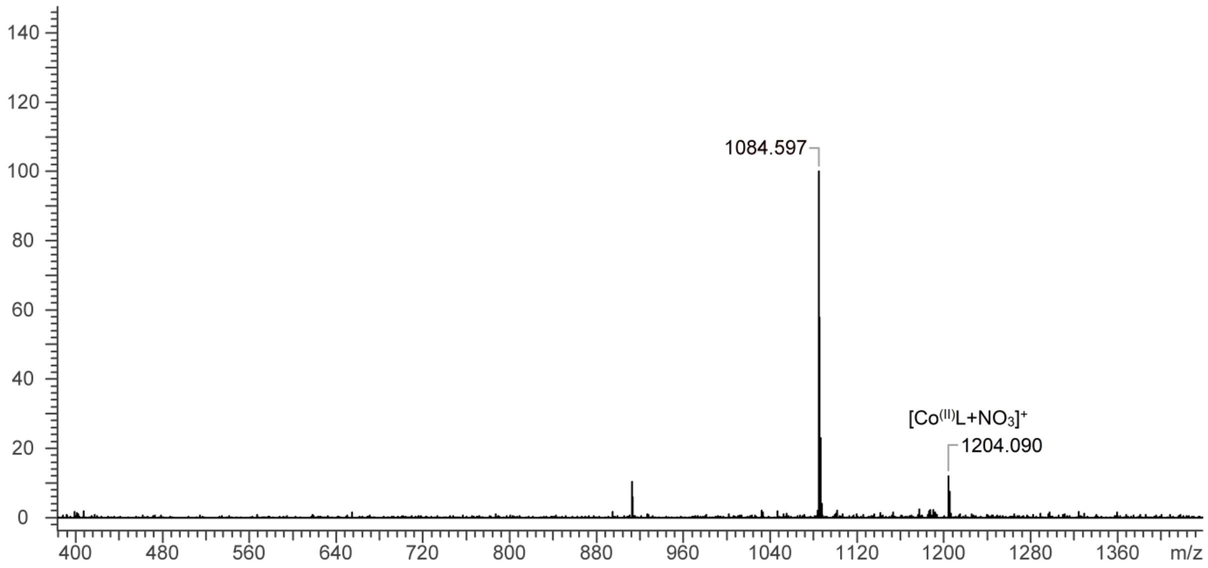

Figure S1. The ESI–mass spectrum of the complex obtained by mixing acetonitrile solutions of L with $Co(NO_3)_2$

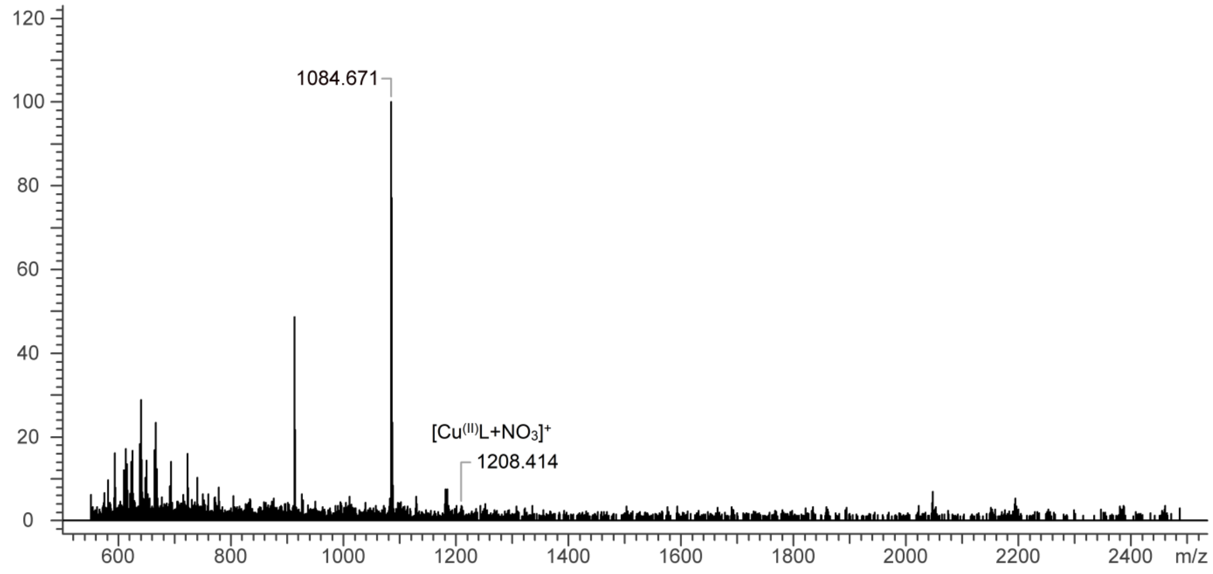

Figure S2. The ESI-mass spectrum of the complex obtained by mixing acetonitrile solutions of L with $Cu(NO_3)_2$

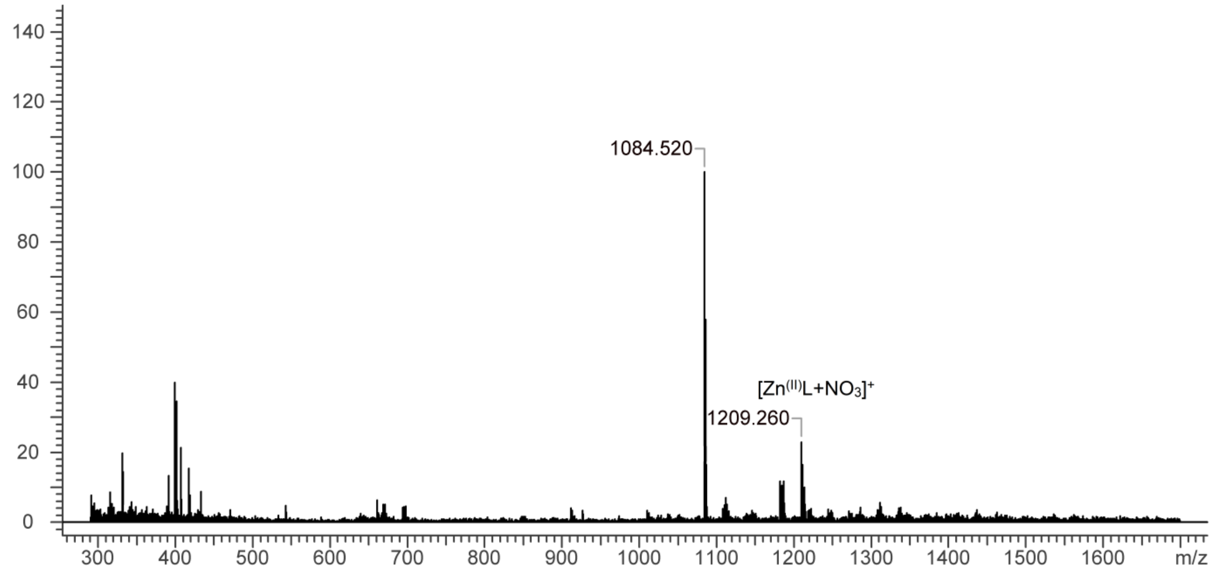

Figure S3. The ESI-mass spectrum of the complex obtained by mixing acetonitrile solutions of L with $Zn(NO_3)_2$

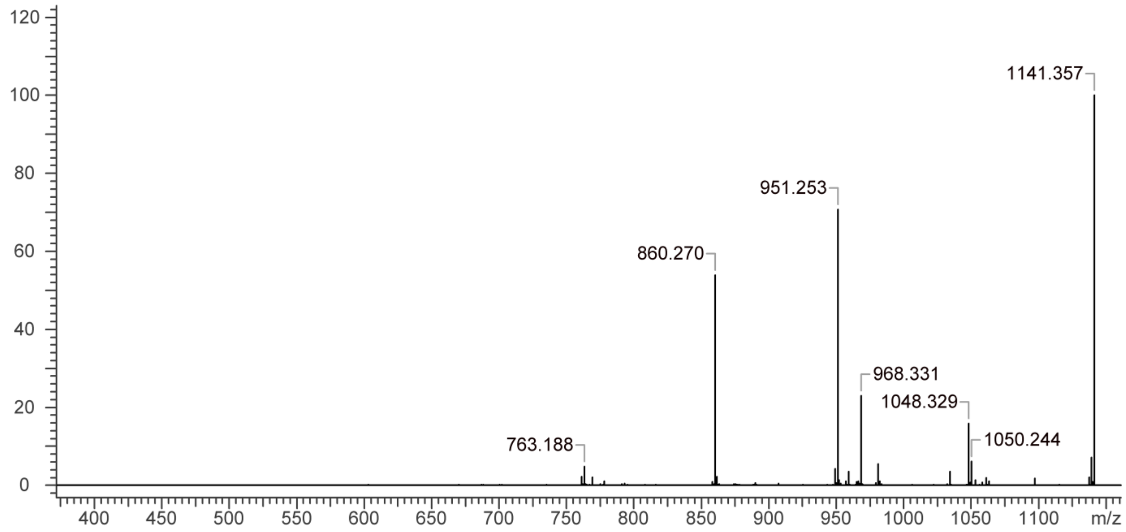

Figure S4. The ESI-MS/MS^2 spectrum of the $[Co(II)L+NO_3]^+$ complex ion at m/z = 1204

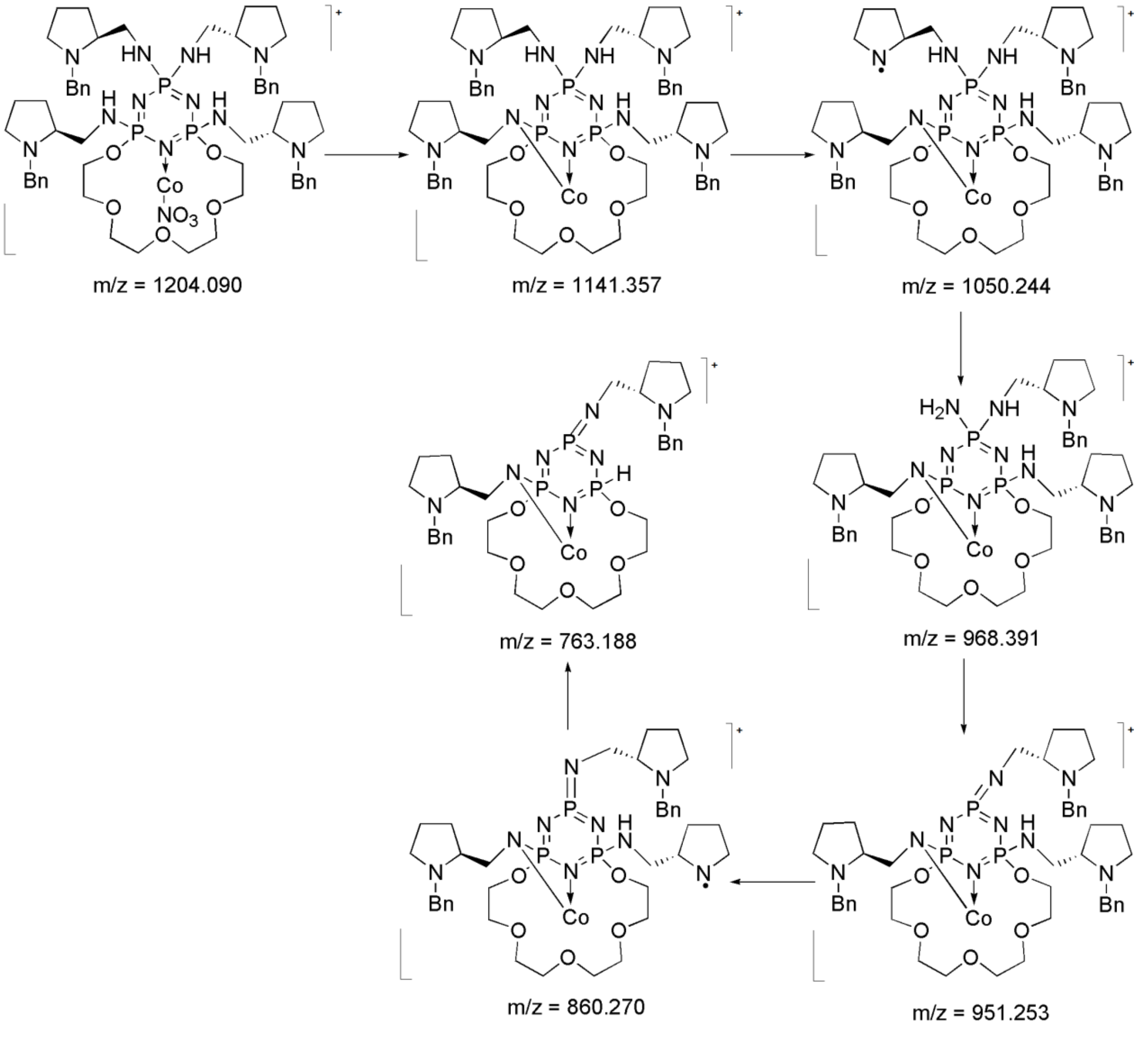

Scheme S1. Fragmentation pathways of the $[Co(II)L+NO_3]^+$ complex ion m/z 1204.

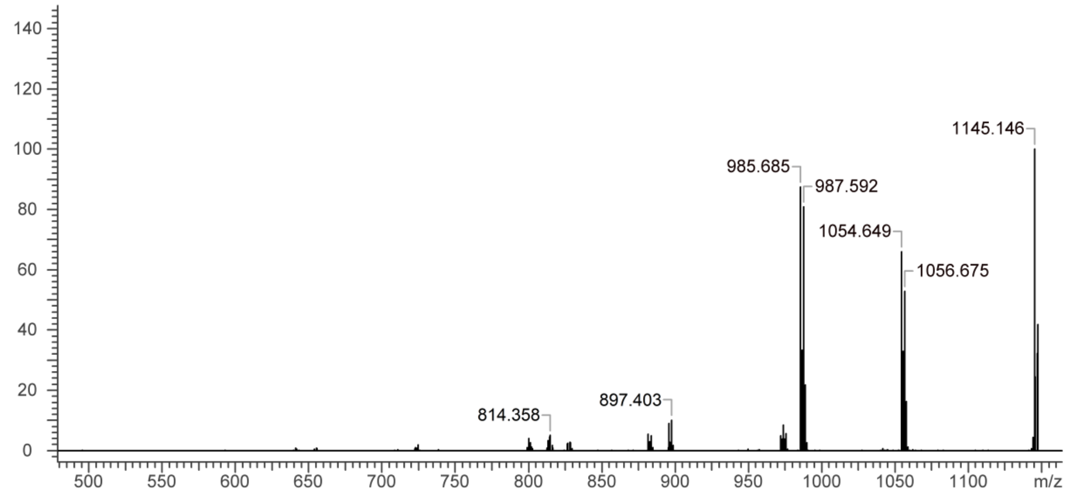

Figure S5. The ESI-MS/MS[2] spectrum of the $[Cu(II)L+NO_3]^+$ complex ion at m/z = 1208

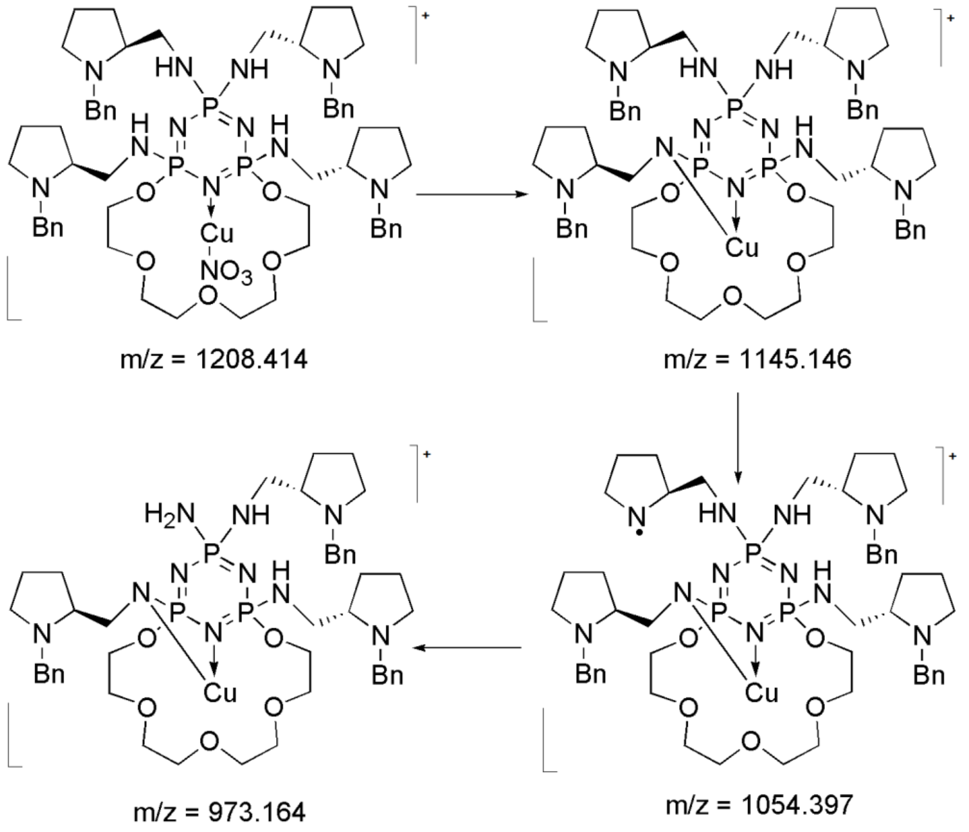

Scheme S2. The first fragmentation pathway of the $[Cu(II)L+NO_3]^+$ complex ion m/z 1208

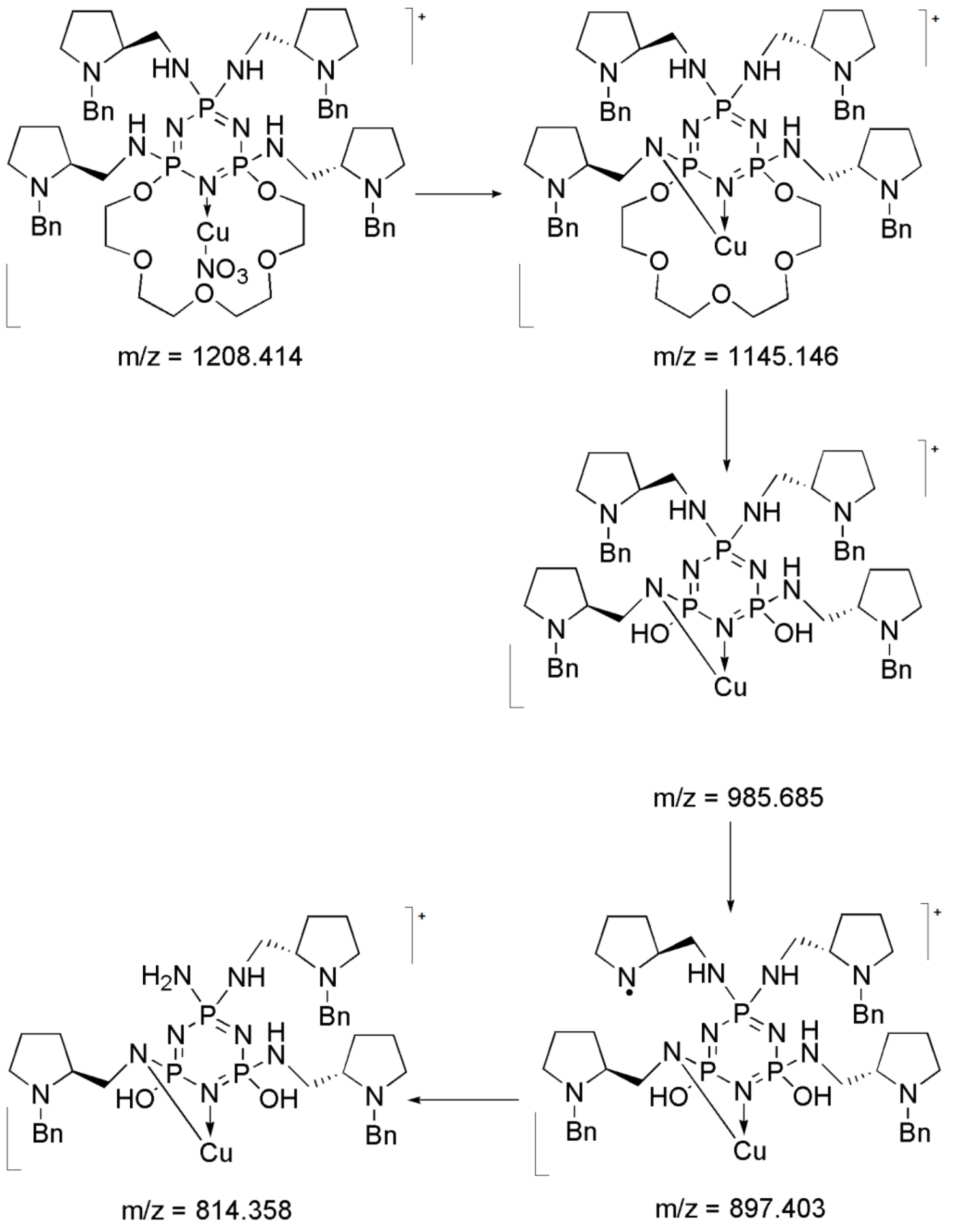

Scheme S3. The second fragmentation pathway of the $[Cu(II)L+NO_3]^{+}$ complex ion m/z 1208.

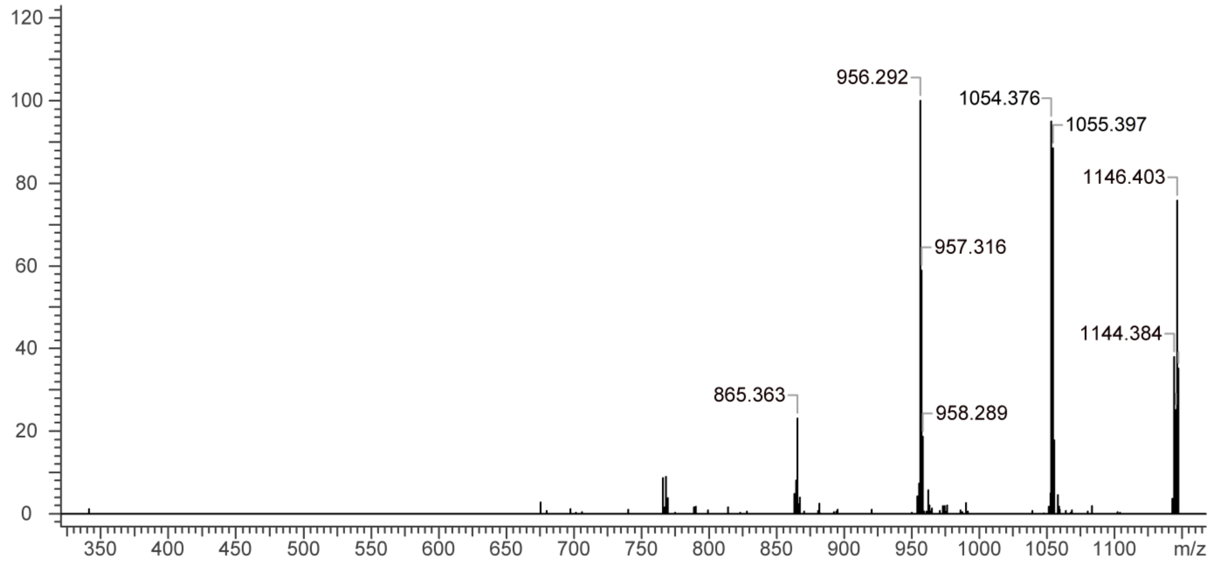

Figure S6. The ESI-MS/MS^2 spectrum of the $[Zn(II)L+NO_3]^+$ complex ion at m/z = 1209

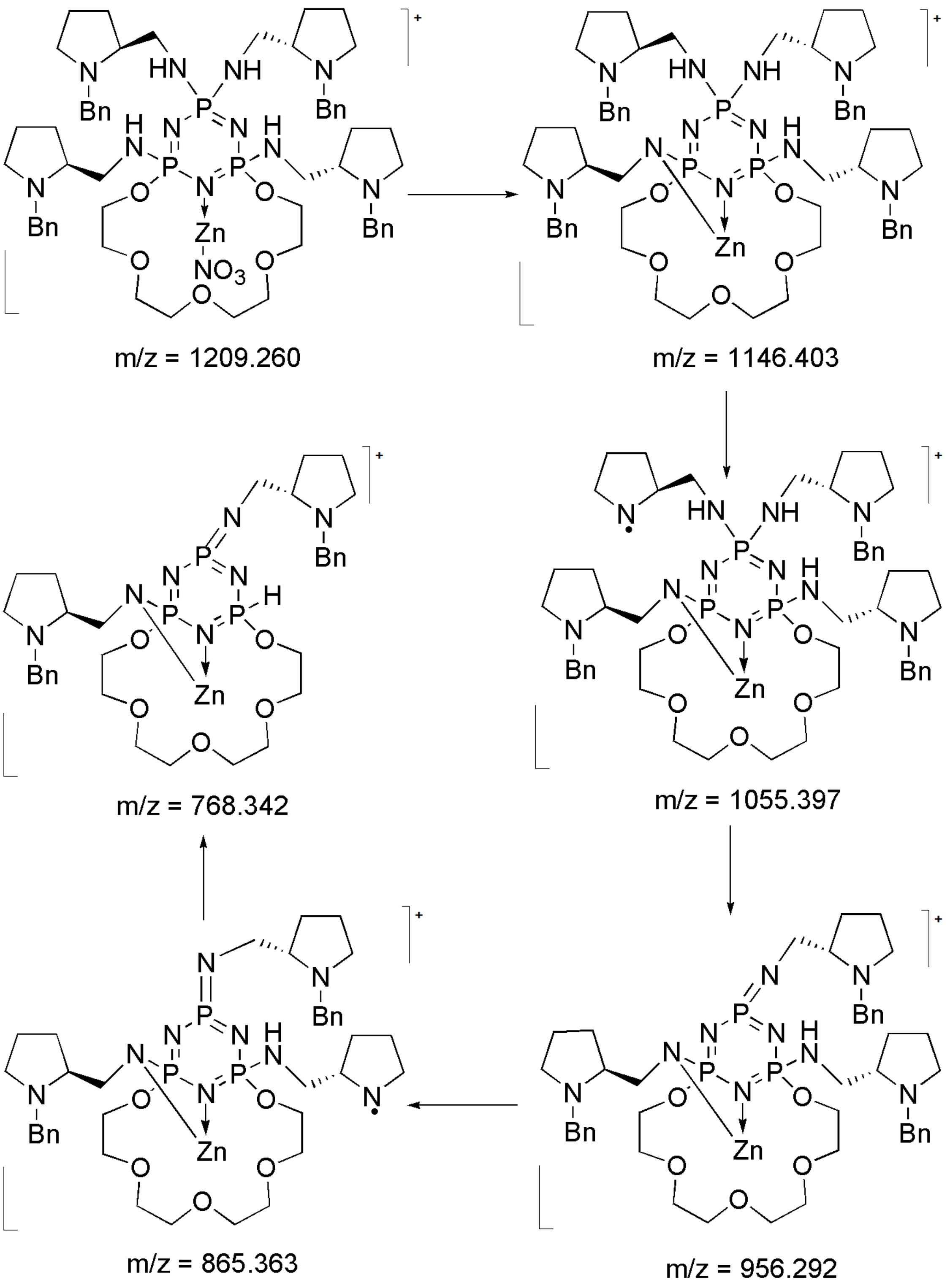

Scheme S4. Fragmentation pathway of the $[Zn(II)L+NO_3]^+$ complex ion m/z .1209.
